# Studies on the Changes in Lipid Peroxidation and Antioxidants in Fishes Exposed to Hydrogen Sulfide

**DOI:** 10.4103/0971-6580.72674

**Published:** 2010

**Authors:** R. Sreejai, D. S. Jaya

**Affiliations:** Department of Environmental Sciences, University of Kerala, Kariavattom Campus, Thiruvananthapuram - 695 581, Kerala, India

**Keywords:** Antioxidants, hydrogen sulfide, lipid peroxidation, malondialdehyde

## Abstract

In the present aquarium study, *Oreochromis mossambicus* Peters were exposed to two different concentrations of hydrogen sulfide (H_2_S) (4.9 and 6.6 mg/l), and the changes in lipid peroxidation (LP) products and antioxidants in test fishes were determined in time intervals of 12, 24, 48, 72, and 96 hours. The results showed that with respect to the H_2_S concentration and duration of exposure, alterations were observed in the concentration of LP products and antioxidants in the various organs of the test fishes. Malondialdehyde (MDA) content increased in the liver, gill, kidney, and brain on exposure to H_2_S up to 48 hours, and then the MDA content showed steady value up to 98 hours experimental period. Brain and kidney of fishes showed the maximum increase in concentration of reduced glutathione (GSH) on H_2_S treatment. The gradual decrease in concentration of GSH in the tissues of H_2_ S-exposed fishes after 48 to 96 hours compared with the control shows the loss of adaptive mechanisms and the oxidation of GSH to glutathione disulphide (GSSG). Slight increase in the activity of GSH-S-transferase and decrease in activity of GSH peroxidase demonstrated the incapability of the vital organs in neutralizing the peroxides generated in the oxidative stress condition.

## INTRODUCTION

Hydrogen sulfide (H_2_S) is a potentially lethal gas produced by anaerobic decomposition of protein and other sulfur-containing organic matter, decomposition of organic effluents from municipal sewage and many industries. It is also formed in the coir retting process usually done in estuaries using coconut husk. It may occur naturally at levels which can be inimical to fish production and survival.[1] The toxic effects of H_2_S are based on its property as a chemical asphyxiate. It binds to the mitochondrial enzyme cytochrome oxidase, blocking oxidative phosphorylation and adenosine triphosphate (ATP) production. This leads to anaerobic metabolism and development of lactic acidosis. Oxidative stress can be defined as an elevation in the steady state concentration of reactive oxygen species, which occur when the balance between the mechanisms triggering oxidative conditions and cellular antioxidant is impaired. The enzymatic and nonenzymatic antioxidant defense mechanisms work together to counter oxidative stress. In this study, an attempt has been made to evaluate the effect of H_2_S exposure at two different concentrations on the fresh water fish, *Oreochromis mossambicus*. The study also aims to find out the oxidative stress in fishes exposed to H_2_S by measuring the concentration of lipid peroxidation (LP) products and the changes in the levels of antioxidants in liver, gill, kidney, and brain tissues.

## MATERIALS AND METHODS

Healthy specimens of *Oreochromis mossambicus* Peters (*Tilapia*) were collected from fish rearing ponds in Thiruvananthapuram and maintained in the laboratory in large glass aquaria containing weathered, well aerated tap water for two weeks. These fishes were treated with potassium permanganate solution (0.5% w/v) in order to get rid of infectious organisms. Acclimation was done at room temperature (28 ± 2°C), and the fishes were exposed to the natural day and night cycle. The fishes were fed with standard food pellets *ad libitum*. The physicochemical characteristics (temperature, dissolved oxygen, pH, total alkalinity, total ammonia nitrogen, and total hardness) of the aquarium water in the tanks were monitored throughout the experimental study period.

H_2_S was prepared by reacting dilute hydrochloric acid with ferrous sulfide sticks in a Kipp’s Apparatus. The gas flow to the aquarium water was regulated by using a flow meter. Exploratory tests were conducted initially to assess the range of concentrations suitable for the diagnostic test. The concentrations of H_2_S used for definitive test in aquarium water were assessed as 4.9 mg/l on two minute passage of the gas and 6.6 mg/l in five minute passage of gas. The lethal concentration of H_2_S for 50% fish death (LC_50_24 hour) was calculated as 6.6 mg/l.

Healthy female fishes of uniform body weight (30 ± 5 g) and body length (12 ± 14 cm) were selected for the study. The fishes were divided into three groups (T_1_, T_2_, and C) with 12 fishes in each group and maintained in the aquarium without feed for 12 hours before exposure to H_2_S. The fishes in T_1_and T_2_groups were experimentally exposed to 4.9 and 6.6 mg/l concentrations of H_2_S respectively under controlled conditions in aquarium water. The third group (C) served as control and these fishes were maintained in tap water free from H_2_S. Two replicate experiments for each test groups were also conducted.

The test and control group fishes were sacrificed after 12, 24, 48, 72, and 92 hours experimental duration and the tissues (liver, gill, kidney, and brain) were collected in ice-cold containers. The tissue homogenates were prepared in appropriate buffers for each estimation. The concentration of the LP product–malondialdehyde (MDA) was determined by the method of Niehaus and Samuelsson.[2] The nonenzymatic antioxidant-reduced glutathione (GSH) was determined by the method of Pattersson and Lazarow,[3] and activities of the enzyme catalase (CAT) was determined by the method of Maehly and Chance.[4] Superoxide dismutase (SOD) was assayed by the method of Kakkar *et al*.,[5] glutathione peroxidase (GPx) was assayed by the method of Habig *et al*.,[6] and glutathione-S-transferase (GST) was determined following the method of Paglia and Valentine.[7] Analysis of important physicochemical characteristics of aquarium water collected initially (before passing H_2_S) and during the experimental period (after different time intervals of the study) was carried out according to standard procedures described in american public health association (APHA).[8] All the biochemicals used for the estimations were purchased from Loba Chemie Pvt. Ltd., India and the other chemicals used in this study were of analytical grade.

### Statistical analysis

All the data are expressed as mean ± standard deviation. Statistical significance of the data were determined by one-way ANOVA (Duncan’s test) using SPSS software, and the results were expressed with significance *P*<0.05 and *P*<0.001.

## RESULTS

### Changes in physicochemical characteristics of water

The physicochemical characteristics of aquarium water with control group and test group fishes are given in Tables 1a–c. The fishes in test groups T_1_ and T_2_ exhibited erratic movements in the water during the experiment. The temperature of water in control group was 26.5 ± 0.58°C, whereas that of the test group varied from 25.75 ± 0.05°C to 28 ± 2.03°C. The H_2_S concentration in control group water was below detectable limit, whereas in T_1_ group water, the H_2_S content after two minute passage was 4.9 ± 0.02 mg/l and then after the 96 hours experimental period, it decreased to 2 ± 0.02 mg/l. In T_2_ group water, the H_2_S content after five minutes passage was 6.6 ± 0.07 mg/l and it decreased to 2.0 ± 0.09 mg/l after 96 hours. The dissolved oxygen content of control group water varied from 6.22 ± 0.09 to 7.39 ± 0.03 mg/l, whereas that of T_1_ group water varied from 2.2 ± 0.01 to 4.2 ± 0.05 mg/l and T_2_ group varied from 1.0 ± 0.06 to 3.8 ± 1.40 mg/l. The pH of the control group water varied from 6.66 ± 0.17 to 6.82 ± 0.05, i.e., almost neutral. The pH of T_1_ group water varied from 6.0 ± 0.12 to 6.4 ± 0.09, whereas that of T_2_ group water varied from 6.0 ± 0.28 to 6.3 ± 0.41, i.e., slightly acidic.

**Table 1a:** Physicochemical characteristics of aquarium water with control group fishes

Parameters	Initial	12 h	24 h	48 h	72 h	96 h
Temperature (°C)	25.25 ± 0.96	26.25 ± 0.5	26.5 ± 0.58	26 ± 1.63	25.75 ± 0.96	25.5 ± 1.23
pH	6.67 ± 0.06	6.67 ± 0.12	6.67 ± 0.12	6.68 ± 0.08	6.68 ± 0.05	6.68 ± 0.17
DO (mg/l)	7.22 ± 0.09	7.39 ± 0.03	7.27 ± 0.04	7.25 ± 0.04	7.29 ± 0.05	7.03 ± 0.16
H_2_S (mg/l)	BDL	BDL	BDL	BDL	BDL	BDL
Sulfate (mg/l)	1.23 ± 0.02	1.25 ± 0.02	1.25 ± 0.02	1.25 ± 0.04	1.23 ± 0.00	1.21 ± 0.05

BDL - below detectable limit; DO - dissolved oxygen; H_2_S - hydrogen sulfide

**Table 1b:** Physicochemical characteristics of aquarium water with T_1_ group fishes

Parameters	2’	12 h	24 h	48 h	72 h	96 h
Temperature (°C)	26.5 ± 0.56	27 ± 0.81	27 ± 0.82	27 ± 0.82	25.75 ± 0.96	25.75 ± 0.05
pH	6.0 ± 0.12	6.2 ± 0.05	6.10.02	6.1 ± 0.01	6.4 ± 0.09	6.3 ± 0.09
DO (mg/l)	2.2 ± 0.01	2.4 ± 0.05	2.8 ± 0.08	3.2 ± 0.12	3.6 ± 012	4.2 ± 0.05
H_2_S (mg/l)	4.9 ± 0.02	4.02 ± 0/04	3.6 ± 0.12	2.8 ± 1.22	2.2 ± 0.08	2.0 ± 0.02
Sulfate (mg/l)	1.53 ± 0.81	1.53 ± 1.12	1.67 ± 0.05	1.70 ± 0.02	1.76 ± 0.52	1.78 ± 1.45

DO - dissolved oxygen; H^2^S - hydrogen sulfide

**Table 1c:** Physicochemical characteristics of aquarium water with T_2_ group fishes

Parameters	5’	12 h	24 h	48 h	72 h	96 h
Temperature (°C)	28 ± 2.03	28 ± 0.07	27 ± 0.06	27 ± 0.22	27 ± 0.15	27 ± 0.08
pH	6.0 ± 0.28	6.1 ± 0.44	6.1 ± 1.45	6.0 ± 1.03	6.2 ± 0.33	6.3 ± 0.41
DO (mg/l)	1.0 ± 0.06	2.4 ± 1.03	2.8 ± 1.21	3.2 ± 1.32	3.6 ± 0.03	3.8 ± 1.40
H_2_S (mg/l)	6.6 ± 0.07	5.8 ± 0.32	4.2 ± 0.44	3.4 ± 0.04	2.6 ± 0.18	2.0 ± 0.09
Sulfate (mg/l)	1.9 ± 0.05	1.9 ± 1.22	1.91 ± 0.06	1.96 ± 0.01	1.99 ± 0.07	1.99 ± 0.08

DO - dissolved oxygen; H_2_S - hydrogen sulfide

### Lipid peroxidative changes in fishes exposed to H_2_S

The changes in the concentration of MDA and antioxidants in the tissues of fishes exposed to H_2_S with respect to the control group are given in Tables 2 – 7.

**Table 2:** Changes in malondialdehyde content in fish tissues

Time interval (Hours)	Malondialdehyde (micromoles/100 g wet tissue)											
	Liver			Gill			Kidney			Brain		
	T1	T2	Control	T1	T2	Control	T1	T2	Control	T1	T2	Control
Initial	219 ± 0.89*	224 ± 6.84*	193 ± 0.09	132 ± 2.82*	132 ± 3.0*	122 ± 1.70	94 ± 5.50*	99 ± 7.27*	84 ± 2.08	48 ± 0.11*	42 ± 2.51*	36 ± 1.63
12	223 ± 0.15*	230 ± 7.11*	193 ± 0.12	140 ± 4.39*	141 ± 5.0*	123 ± 1.03	98 ± 4.72*	108 ± 9.88*	84 ± 1.29	51 ± 0.26*	51 ± 3.82*	35 ± 1.71
24	231 ± 0.04*	238 ± 7.27*	193 ± 0.0	142 ± 4.03*	145 ± 5.77*	123 ± 1.26	103 ± 3.0*	113 ± 8.99*	84 ± 2.16	51 ± 0.19*	53 ± 3.41*	36 ± 1.70
48	232 ± 0.16*	239 ± 0.14*	193 ± 0.56	143 ± 1.73*	144 ± 0.01*	124 ± 0.95	101 ± 2.5*	122 ± 0.01*	84 ± 1.73	49 ± 1.91*	51.25 ± 3.59*	37 ± 1.15
72	232 ± 0.06*	239 ± 0.12*	193 ± 0.96	143 ± 3.78*	143 ± 0.02*	124 ± 0.96	101 ± 0.08*	122 ± 0.01*	85 ± 1.0	49 ± 0.23*	51 ± 0.02	35 ± 1.25
96	232 ± 0.07*	239 ± 1.90*	193 ± 0.48	143 ± 0.04*	144 ± 0.10*	124 ± 0.96	101 ± 0.02*	122 ± 0.02*	84 ± 2.06	49 ± 0.02*	51 ± 0.01	35 ± 1.29

Values are mean ± S.D, No of fishes = 6;

* *P*<0.05; SD - standard deviation

**Table 3:** Changes in glutathione (GSH) content in fish tissues

Time interval (Hours)	Reduced glutathione (nanomoles/100 f wt)											
	Liver			Gill			Kidney			Brain		
	T1	T2	Control	T1	T2	Control	T1	T2	Control	T1	T2	Control
0	1266 ± 8.06*	1272 ± 3.56*	1250 ± 6.27	1122 ± 0.29*	1125 ± 4.43*	1058 ± 1.63	1326 ± 1.32*	1375 ± 2.94*	1288 ± 0.95	948 ± 0.95*	1104 ± 2.64*	620 ± 0.58
12	1272 ± 7.11**	1286 ± 1.70*	1250 ± 7.04	1221 ± 4.96*	1222 ± 0.95*	1058 ± 1.0	1382 ± 2.64*	1425 ± 2.5*	1290 ± 0.95	995 ± 6.07*	1105 ± 2.22*	622 ± 1.70
24	1273 ± 3.82*	1304 ± 3.65*	1251 ± 5.48	1222 ± 129	1246 ± 2.70*	1059 ± 1.29	1363 ± 3.5*	1456 ± 2.87**	1292 ± 1.5	988 ± 5.91*	1124 ± 1.71*	620 ± 0.5
48	1262 ± 4.90	1320 ± 2.38	1250 ± 6.78	1225 ± 2.58*	1245 ± 1.63	1060 ± 2.16	1459 ± 1.70	1535 ± 1.91**	1291 ± 1.29	865 ± 6.45*	983 ± 1.26*	619 ± 1.29
72	1260 ± 0.17	1299 ± 0.37	1251 ± 5.61	1226 ± 1.29	1246 ± 1.82*	1060 ± 3.0	1553 ± 2.5	1574 ± 1.71	1292 ± 1.5	854 ± 1.73*	982 ± 2.38**	620 ± 0.57
96	1260 ± 0.17	1298 ± 0.06	1251 ± 2.06	1225 ± 1.23	1251 ± 1.89	1061 ± 2.38	1554.3 ± 1.25	1574 ± 2.23	1291 ± 3.10	851 ± 1.71*	981 ± 0.82*	620 ± 1.82

Values are mean ± S.D, No of fishes = 6;

* *P*<0.05;

** *P*<0.001; SD - standard deviation

**Table 4:** Changes in superoxide dismutase activity in fish tissues

Time interval (Hours)	Superoxide dismutase activity (Units/mg protein)											
	Liver			Gill			Kidney			Brain		
	T1	T2	Control	T1	T2	Control	T1	T2	Control	T1	T2	Control
0	9.19 ± 0.12*	10.21 ± 0.13*	6.86 ± 0.12	2.75 ± 0.39*	2.74 ± 0.50*	1.74 ± 0.45	9.27 ± 0.11**	9.93 ± 0.02*	5.58 ± 0.57	0.72 ± 0.05*	0.84 ± 0.13*	0.43 ± 0.01
12	9.31 ± 0.07*	10.67 ± 0.42*	6.78 ± 0.14	3.14 ± 0.34**	3.17 ± 0.06**	1.68 ± 0.07	9.48 ± 0.31*	10.65 ± 0.02*	5.54 ± 0.01	0.75 ± 0.03*	0.86 ± 0.16*	0.42 ± 0.00
24	8.72 ± 0.06*	9.78 ± 0.14*	6.75 ± 0.17	2.14 ± 0.16	2.70 ± 0.49*	1.72 ± 0.05	7.47 ± 0.23*	8.40 ± 0.32*	5.352 ± 0.01	0.62 ± 0.01*	0.72 ± 0.09*	0.42 ± 0.01
48	7.55 ± 0.04*	7.74 ± 0.03*	6.56 ± 0.36	1.92 ± 0.03*	2.13 ± 0.02*	1.60 ± 0.15	7.25 ± 0.02*	7.81 ± 0.02*	5.41 ± 0.10	0.53 ± 0.01*	0.62 ± 0.10*	0.41 ± 0.01
72	7.43 ± 0.15*	7.64 ± 0.04*	6.63 ± 0.40	1.91 ± 0.02	2.12 ± 0.04	1.63 ± 0.17	6.84 ± 0.28*	7.54 ± 0.01*	5.46 ± 0.16	0.52 ± 0.01	0.58 ± 0.05	0.42 ± 0.01
96	7.41 ± 0.12	7.62 ± 0.06	6.28 ± 0.50	1.85 ± 0.20	2.06 ± 0.01	1.57 ± 0.21	6.72 ± 0.04	7.5 ± 0.01	5.38 ± 0.13	0.52 ± 0.01	0.58 ± 0.06	0.42 ± 0.01

Values are Mean ± S.D, No of fishes = 6;

* *P*<0.05;

** *P*<0.001; SD - standard deviation

**Table 5:** Changes in catalase activity in fish tissues

Time interval (Hours)	Catalase activity (micromoles of H_2_O_2_ decomposed /mg protein/minute)											
	Liver			Gill			Kidney			Brain		
	T1	T2	Control	T1	T2	Control	T1	T2	Control	T1	T2	Control
0	11.32 ± 0.87	12.25 ± 0.33	10.19 ± 0.15	13.09 ± 0.37*	13.29 ± 0.55**	12.29 ± 0.31	8.74 ± 1.189**	9.32 ± 0.63**	7.33 ± 0.14	6.98 ± 0.01**	7.00 ± 0.21**	5.79 ± 0.59
12	11.50 ± 0.92**	12.27 ± 0.24**	10.19 ± 0.13	12.91 ± 0.45**	13.54 ± 0.49*	12.29 ± 0.31	8.92 ± 1.026**	9.50 ± 0.48**	7.36 ± 0.15	6.78 ± 0.162*	7.08 ± 0.20**	5.78 ± 0.60
24	11.32 ± 0.96*	11.61 ± 0.75**	10.18 ± 0.11	12.86 ± 0.72**	13.20 ± 0.30**	12.27 ± 0.29	9.21 ± 0.926*	9.20 ± 1.00*	7.34 ± 0.14	6.85 ± 0.39**	7.06 ± 0.11**	5.78 ± 0.59
48	10.62 ± 1.03**	10.79 ± 0.63**	10.18 ± 0.99	11.86 ± 0.03	12.46 ± 0.019**	12.30 ± 0.31	7.86 ± 0.026**	7.64 ± 0.032**	7.36 ± 0.15	6.265 ± 0.124**	6.66 ± 0.22**	5.75 ± 0.60
72	9.98 ± 0.840**	10.13 ± 0.33**	10.19 ± 0.55	11.8 ± 0.77	12.45 ± 0.35	12.29 ± 0.31	7.79 ± 0.019**	7.61 ± 0.024	7.34 ± 0.14	6.22 ± 0.012	6.62 ± 0.033**	5.74 ± 0.61

Values are Mean ± S.D, No of fishes = 6;

* *P*<0.001;

** *P*<0.05; SD - standard deviation

**Table 6:** Changes in glutathione peroxidase activity in fish tissues

Time interval (Hours)	Glutathione peroxidase activity (micromoles of NADH oxidised /mg protein/min)											
	Liver			Gill			Kidney			Brain		
	T1	T2	Control	T1	T2	Control	T1	T2	Control	T1	T2	Control
0	6.8 ± 0.08*	7.63 ± 0.847*	5.84 ± 0.149	3.64 ± 0.462*	3.5 ± 0.52*	2.97 ± 0.01	1.00 ± 0.171*	1.05 ± 0.10*	0.59 ± 0.01	0.29 ± 0.01*	0.195 ± 0.023*	0.107 ± 0.027
12	6.90 ± 0.394*	7.99 ± 0.842*	5.84 ± 0.164	3.69 ± 0.44*	3.52 ± 0.52*	2.97 ± 0.02	1.09 ± 0.01**	1.08 ± 0.018*	0.59 ± 0.01	0.295 ± 0.04*	0.217 ± 0.029*	0.112 ± 0.030
24	6.93 ± 0.23*	6.82 ± 0.633	5.84 ± 0.153	3.44 ± 0.275*	3.44 ± 0.02*	2.97 ± 0.02	1.05 ± 0.01*	0.99 ± 0.02*	0.59 ± 0.01	0.242 ± 0.427*	0.195 ± 0.034*	0.122 ± 0.027
48	6.35 ± 0.02*	6.76 ± 0.561*	5.84 ± 0.15	3.09 ± 0.15	3.03 ± 0.01	2.97 ± 0.02	1.085 ± 0.02*	0.78 ± 0.01	0.58 ± 0.03	0.19 ± 0.012*	0.15 ± 0.02*	0.11 ± 0.08
72	6.33 ± 0.018*	6.78 ± 0.02*	5.84 ± 0.01	3.02 ± 0.012	2.93 ± 0.49	2.96 ± 0.01	0.84 ± 0.02*	0.77 ± 0.015	0.58 ± 0.017	0.187 ± 0.047*	0.142 ± 0.017*	0.115 ± 0.02
96	6.32 ± 0.01*	6.76 ± 0.02*	5.84 ± 0.03	3.022 ± 0.01	2.93 ± 0.01	2.97 ± 0.01	0.83 ± 0.01	0.76 ± 0.012	0.58 ± 0.03	0.185 ± 0.01	0.145 ± 0.01	0.1225 ± 0.017

Values are Mean ± S.D, No of fishes = 6;

* *P*<0.05;

** *P*<0.001; NADH - nicotinamide adenine -dinucleotide, reduced; SD - standard deviation

**Table 7:** Changes in glutathione S-transferase activity in fish tissues

Time interval (Hours)	Glutathione S-transferase activity (micromoles of thioether formed /mg protein/min)											
	Liver			Gill			Kidney			Brain		
	T1	T2	Control	T1	T2	Control	T1	T2	Control	T1	T2	Control
0	3.77 ± 0.17*	3.88 ± 0.53*	3.05 ± 0.02	0.822 ± 0.08*	0.91 ± 0.07*	0615 ± 0.02	3.005 ± 0.64*	3.062 ± 0.10*	2.11 ± 0.01	0.24 ± 0.04*	0.33 ± 0.02*	0.172 ± 0.05
12	3.77 ± 0.37*	3.98 ± 0.60*	3.05 ± 0.04	0.86 ± 0.08**	0.942 ± 0.05*	0.617 ± 0.01	3.05 ± 0.64*	3.305 ± 0.24*	2.12 ± 0.01	0.26 ± 0.02*	0.37 ± 0.02*	0.175 ± 0.39
24	3.55 ± 0.39*	3.95 ± 0.58*	3.06 ± 0.04	0.85 ± 0.04*	0.89 ± 0.04*	0.622 ± 0.00	3.12 ± 0.75**	3.365 ± 0.18*	2.11 ± 0.02	0.26 ± 0.03*	0.33 ± 0.01*	0.167 ± 0.04
48	3.18 ± 0.05*	3.28 ± 0.00*	3.05 ± 0.02	0.747 ± 0.00*	0.732 ± 0.02*	0.617 ± 0.01	2.47 ± 0.06*	2.64 ± 0.03**	2.31 ± 0.05	0.19 ± 0.01*	0.25 ± 0.01*	0.17 ± 0.05
72	3.17 ± 0.03*	3.12 ± 0.02*	3.06 ± 0.02	0.722 ± 0.05*	0.722 ± 0.01*	0.627 ± 0.00	2.36 ± 0.03*	2.64 ± 0.18*	2.12 ± 0.01	0.17 ± 0.03*	0.23 ± 0.02*	0.172 ± 0.04
96	3.14 ± 0.02	3.09 ± 0.005	3.06 ± 0.04	0.710 ± 0.00*	0.702 ± 0.01	0.620 ± 0	2.34 ± 0.03	2.487 ± 0.02*	2.13 ± 0.03	0.17 ± 0.01*	0.21 ± 0.02*	0.178 ± 0.06

Values are Mean ± S.D, No of fishes = 6;

* *P*<0.05;

** *P*<0.001; SD - standard deviation

Changes in LP lead to destruction of membrane lipids and production of lipid peroxides and their byproducts such as aldehydes. The MDA content in liver of the fishes in T_1_ group (48 hours) was 232 ± 0.16 *μ* moles/100 g wet tissue and in T_2_ group (48 hours), it was 239 ± 0.90 *μ* moles/100 g wet tissue compared with that of the control fishes. In gills, the MDA content was recorded as 143 ± 3.78 *μ* moles/100 g wet tissue in T_1_ group (48 hours) and 145 ± 5.77 *μ* moles/100 g wet tissue in T_2_ group, compared with that of the control group (122 ± 1.70 *μ* moles/100 g wet tissue). The kidney MDA content showed a significant increase to 103 ± 3.0 *μ* moles/100 g wet tissue in T_1_ group (48 hours) and to 122 ± 0.02 *μ* moles/100 g wet tissue in T_2_ group (48 hours) compared with that of the control group, i.e., 84 ± 1.29 *μ* moles/100 g wet tissue. In the brain tissue of T_1_ group (48 hours), the MDA content was estimated to be 51 ± 0.26 *μ* moles/100 g wet tissue and in T_2_ group (48 hours), it was 53 ± 3.41 *μ* moles/100 g wet tissue, which were significantly higher (*P*<0.05) than their respective healthy controls. After 48 hours, the MDA values in different tissues remain constant up to the end of the experimental period (96 hours), and it was also observed that the H_2_S concentration in aquarium water also decreased with time.

In the present investigation, liver, gill, and brain showed time- and dose-dependent changes in GSH level. There was initial elevation in GSH level, but in the low and medium lethal concentrations of H_2_S-exposed fishes, there was a slight decrease in GSH level in the liver and brain toward the end of the experiment (96 hours). In liver, the GSH content varied from 1250 ± 6.27 (C) to 1273 ± 3.82 nm/100 f wt (T_1_-24 hours) and 1320 ± 2.38 nm/100 f wt (T_2_-48 hours) and in gills, it increased from 1058 ± 1.63 (C) to 1226 ± 1.29 nm/100 f wt (T_1_-72 hours) and 1251 ± 1.89 (T_2_-72 hours). In the kidney of fishes, GSH content increased from 1288 ± 0.95 (C) to 1554 ± 1.25 nm/100 f wt (T_1_-96 hours) and 1574 ± 2.23 nm/100 f wt (T_2_-96 hours) on H_2_S exposure. In brain, it varied from 619 ± 1.29 (C) to 995 ± 6.07 nm/100 f wt (T_1_-12 hours) and 1124 ± 1.71 nm/100 f wt (T_2_-48 hours) in H_2_S-exposed fishes. A significant initial increase and gradual decrease in GSH level was observed in liver and brain, but in kidney and gills, it showed gradual elevation in concentration when the fishes are kept in H_2_S contaminated water for 96 hours.

The activity of SOD in liver tissues of *Oreochromis mossambicus* recorded 6.28 ± 0.50 units in control and in T_1_and T_2_ groups, the activity showed 9.31 ± 0.07 units (12 hours) and 10.67 ± 0.42 units (12 hours), respectively. In gills, the SOD activities varied between 1.57 ± 0.21 units (C) to 3.14 ± 0.34 units (12 hours) and 3.17 ± 0.56 units (12 hours) in T_1_ and T_2_ groups. In kidney, it varied from 5.352 ± 0.01 units (C) to 9.48 ± 0.31 units (T_1_-12 hours) and 10.65 ± 0.02 units (T_2_-12 hours) and in brain, it varied from 0.41 ± 0.01 units (C) to 0.75 ± 0.03 units (T_1_-12 hours) and 0.86 ± 0.16 units (T_2_-12 hours) on H_2_S exposure. In T_1_ and T_2_ group fishes, all tissues shows significantly increased values (*P*<0.05) compared with that of the healthy control fishes.

The liver CAT activity increases in H_2_S-treated test groups (T_1_ and T_2_) and was recorded as 11.50 ± 0.926 units (T_1_-12 hours) and 12.27 ± 0.24 units (T_2_-12 hours), respectively compared with that 10.18 ± 0.09 units of the control group. Similar increase was also observed in other tissues of the test groups studied. The activity of CAT in the tissues of test group fishes further increased up to 24 hours and then there was a slight decrease in CAT activity in liver, gills, kidney, and brain of fishes after 72 hours up to 96 hours H_2_S exposure.

GPxs are regarded as important component of the cellular system defense against oxidative stress. In the present study, a sharp, duration-dependent decrease in GPx activity level was recorded in all tissues. The GPx activity in liver increases from 5.84 ± 0.01 units (C) to 6.93 ± 0.23 units (T_1_-24 hours) and 7.99 ± 0.842 units (T_2_-12 hours) and in gills, 2.96 ± 0.01 units (C) to 3.52 ± 0.52 units (T_2_-12 hours) and 3.69 ± 0.44 units (T_1_-12 hours). In kidney, the GPx activity recorded was 0.58 ± 0.01 units in control group and test groups showed 1.08 ± 0.10 units (T_2_-12 hours) and 1.09 ± 0.01 units (T_1_-12 hours). In brain, the GPx activity varied from 0.107 ± 0.027 units (C) to 0.217 ± 0.029 units (T_2_-12 hours) and 0.295 ± 0.04 units (T_1_-12 hours).

GST is an enzyme involved in the detoxification and conjugation of xenobiotics and in protecting against peroxidative damage. In this study, GST showed a duration-dependent elevation in GST activity. The GST activity in the liver of fish samples varied from 3.05 ± 0.02 units (C) to 3.77 ± 0.37 units (T_1_-12 hours) and 3.98 ± 0.60 units (T_2_-12 hours), and in gills from 0.615 ± 0.02 units (C) to 0.86 ± 0.08 units (T_1_-12 hours) and 0.942 ± 0.05 units (T_2_-12 hours). In kidney, GST activity varied from 2.11 ± 0.01 units (C) to 3.12 ± 0.75 units (T_1_-24 hours) and 3.36 ± 0.18 units (T_2_-24 hours) and in the brain, it varied from 0.167 ± 0.04 units in control group to 0.26 ± 0.03 units (T_1_-24 hours) and 0.37 ± 0.02 units (T_2_-12 hours) in test group fishes. On exposure of test group fishes to H_2_S for 96 hours, all the four tissues studied showed inhibition in GST activity.

## DISCUSSION

The physicochemical characteristics of the H_2_S-exposed aquarium water showed that there was a considerable decrease in dissolved oxygen content and pH, which leads to chronic oxidative stress in fishes. In water, the H_2_S dissociates, forming monohydrogensulfide and sulfide ions. The relative concentrations of these species are a function of the pH of the water. In aerated water, H_2_S is readily oxidized to sulfates and biologically oxidized to elemental sulfur. The conversion of H_2_S into sulfate and sulfides cause extra pressure to the fishes.[9]

H_2_S is a potent inhibitor of aerobic respiration. H_2_S exposure causes oxidative stress in fishes and results in LP. LP leads to destruction of membrane lipids and production of lipid peroxides and their by-products such as aldehydes. MDA is formed from the breakdown of polyunsaturated fatty acids and it serves as a convenient index for determining the extent of LP.[10] It can be considered as a biomarker of effect representing the state of membrane LP. In the present investigation, liver, kidney, and gill of the fishes subjected to different concentrations of H_2_S exhibited elevated MDA level, which was both time- and dose-dependent. Studies also showed similar increase in MDA content in the muscle and gill tissues of Oreochromis niloticus exposed to high concentrations of etoxazole in long-term durations.[11] In the present experimental study, kidney of test fishes showed the highest MDA content compared with that of control samples. This might be due to the elevated oxidation of molecular oxygen (O_2_) to produce superoxide radicals, indicating the importance of kidney in the detoxification process. This reaction could also be the source of hydrogen peroxide (H_2_O_2_), which caused the production of MDA by initiating the peroxidation of unsaturated fatty acids in the membrane. The activities of the endogenous enzymes to remove the continuously generated free radicals initially increase due to induction but later enzyme depletion occurs, resulting in oxidative cell damage. In the case of environmental exposure of fishes in surface waters to H_2_S by different anthropogenic activities occasionally, the generation of reactive free radicals overwhelms the function of antioxidant defence mechanisms and LP of the cell membrane occurs. Therefore, this causes disturbances in cell integrity and might lead to cell damage/death.[12]

GSH is a sulfhydryl antioxidant, antitoxin, and enzyme co-factor. It is involved in many process including protein and DNA synthesis, xenobiotics conjugation, and antioxidant protection. Although there was a significantly high (*P*<0.05) initial elevation in GSH level, the activity of this enzyme decreased significantly in liver, gill, kidney, and brain (*P*<0.001). Increased GSH level could be an adaptive mechanism to slight oxidative stress, but decreased GSH level could be due to loss of adaptive mechanisms and the oxidation of GSH to GSSG (oxidized GSH). When fish tissues are in contact with the toxicant, these were removed by conjugation with GSH directly or by means of GSTs, which decreased GSH levels. In addition, the oxidative damage caused by metabolites of the toxicant could be mediated by uncoupling of mitochondrial oxidative phosphorylation.[13]

SOD is one of the key enzymes that provide the first line defense against the pro-oxidants and catalyses the transformation of superoxide radicals to H_2_O_2_ and O_2_.[14] Toxic stress is known to alter the activity of SOD in the vital tissues of fish. In the present study, the sublethal and medium lethal concentrations of H_2_S exposure to fishes show an initial elevation in SOD activity up to 24 hours, followed by reduction towards the end of the experimental period. The initial increase in SOD activity indicated the generation of superoxide radical anion, and the inhibition at the end might be due to the higher amount of oxyradical formation than that could be neutralized by the enzyme. It has also been reported in some cases that the superoxide radical by itself or after its transformation to H_2_O_2_caused a strong oxidation of the cysteine in the enzyme and decrease in the SOD activity.[15]

CAT belongs to the cellular antioxidant system that counteracts the toxicity of reactive oxygen species (ROS). They are the heme-containing enzymes that facilitate the removal of H_2_O_2_, which is metabolized to O_2_and water. In the present investigation, CAT activity was significantly (*P*<0.05) increased at the initial phase. A pro-oxidant condition elicited by the presence of toxicant could be triggering an increase in the activity of this antioxidant enzyme at the initial stages of exposure as an adaptive response.[16] A significant decrease (*P*<0.05) in activity of CAT was observed in 96-hours experiment. The low levels of CAT could be attributed to high production of superoxide anion radical.[17]

Peroxidases are enzymes that reduce a variety of peroxides to their corresponding alcohols. Glutathione peroxide is considered to play an important role in protecting membranes from damage due to LP. This observation led to the view that the major detoxification function of GPx is the termination of radical chain propagation by quick reduction to yield further radicals.[18] GPxs are regarded as important components of the cellular system of defense against oxidative stress resulting from the metabolism of xenobiotics.[19] In the present experiment, a significantly (*P*<0.001) sharp duration-dependant decrease in GPx activity level was recorded in highest duration of exposure. The low activity of GPx in different tissues of exposed fish demonstrated the incapability of these organs in neutralizing the impact of peroxides.[20] The reduction of GPx activity in various tissues in the H_2_S-exposed fish might be attributed to the longer influence of various organic and inorganic redox active contaminants.[21] The decreased level of GPx in the H_2_S intoxicated fish might weaken the antioxidant defense system of the fish which would eventually affect their survival.

GST, an enzyme involved in the detoxification and conjugation of xenobiotics and in protecting against peroxidative damage, is ubiquitous in the cytosol and microsomes of eukaryotes. Significant (*P*<0.05) duration-dependent elevation in GST activity was noted in the tissues of *Oreochromis mossambicus* intoxicated with the low and intermediate sublethal concentrations of H_2_S in the present experiment. GST-mediated conjugation might be an important mechanism for detoxifying preoxidised lipid breakdown products, which have a number of adverse biological effects when present in higher amounts. Induced GST activity indicated the role of this enzyme in protection against the toxicity of xenobiotics.[22] In the present study, at the highest sublethal concentration, all the four tissues of the test fishes exhibited inhibition in GST activity. These results are in relation with the studies reported in the Egyptian catfish—*Clarias lazera* subjected to dimethoate exposure, and the study showed strong inhibition of GST in the exposed fish.[23] The reduction in GST activity noted in fish tissues at the highest exposure time of H_2_S indicated the impaired detoxification mechanism of the fish under long-term exposure.

The observations from the present study showed that H_2_S at sublethal and medium lethal concentrations altered the rate of LP and activities of antioxidant systems in various organs of the test fishes. This study on lipid peroxidative changes in *Oreochromis mossambicus* in H_2_S contaminated water shows that changes in antioxidant enzyme activities in fishes plays an important role in the quality assessment of the H_2_S-polluted aquatic medium in which they survive and also for monitoring the fish health in coir retting areas of estuaries.
